# Human Ocular Thelaziasis: A Case Report

**DOI:** 10.31729/jnma.6447

**Published:** 2021-10-31

**Authors:** Anjila Pal, Alok Atreya, Nabina Maharjan, Monika Mahat, Rabin Bom

**Affiliations:** 1 Department of Microbiology, Lumbini Medical College, Palpa, Nepal; 2 Department of Forensic Medicine, Lumbini Medical College, Palpa, Nepal; 3 Department of Ophthalmology, Lumbini Medical College, Palpa, Nepal; 4 Department of General Practice & Emergency Medicine, Maharajgunj Medical Campus, Institute of Medicine, Kathmandu, Nepal

**Keywords:** *Nepal*, *ocular infection*, *Thelazia*, *zoonoses*

## Abstract

Thelaziasis is a zoonotic disease which affects the eye of domestic and wild carnivores caused by the nematode Thelazia. It is transmitted to humans by secretophagous arthropod-borne zoophilic nonbiting flies of the family Drosophilidae. Human thelaziasis is rare and occurs in poor socio-economic families of the rural locations where people live in close proximity with animals. A one and halfyear-old was presented to the outpatient ophthalmology clinic after her mother noticed a whitish, thread-like worm in her right eye. A total of four worms were mechanically removed from her right eye. All the collected worms were gravid female nematodes of Thelazia species. The present case of human ocular thelaziasis from Palpa, Nepal is presented for its rarity.

## INTRODUCTION

Ocular diseases and resulting blindness are traumatic incidents that have the potential of deteriorating the quality of life. Thelaziasis is an ocular infestation caused by the nematode of the genus Thelazia that infects conjunctival sac, lacrimal duct, and lacrimal gland.^1^ The definitive host of this parasite are domestic and wild carnivores (dogs, cats, foxes, and wolves) however, on rare occasions human infestations is also reported.^2,3^ Human thelaziasis predominately occurs in rural communities where people have poor living standards with close proximity to animals.^4^ We report a rare case of human ocular thelaziasis in one and halfyear-old female child from Nepal.

## CASE REPORT

During the first week of August, the patient was brought to the Department of Ophthalmology after her mother noticed a whitish, thread-like worm in the conjunctiva of the right eye. The mother reported that the child slept with her eyes half-closed. On examination, it was noted that the child rubbed her right eye frequently. No purulent discharge or trauma was noted. Visual acuity and slit-lamp examination were normal and did not reveal corneal abrasions or hypopyon. The worms were extracted from her right superior palpebral conjunctiva using a cotton swab. A total of 4 worms were removed and were sent for identification to the Clinical Microbiology Laboratory.

**Figure 1:**
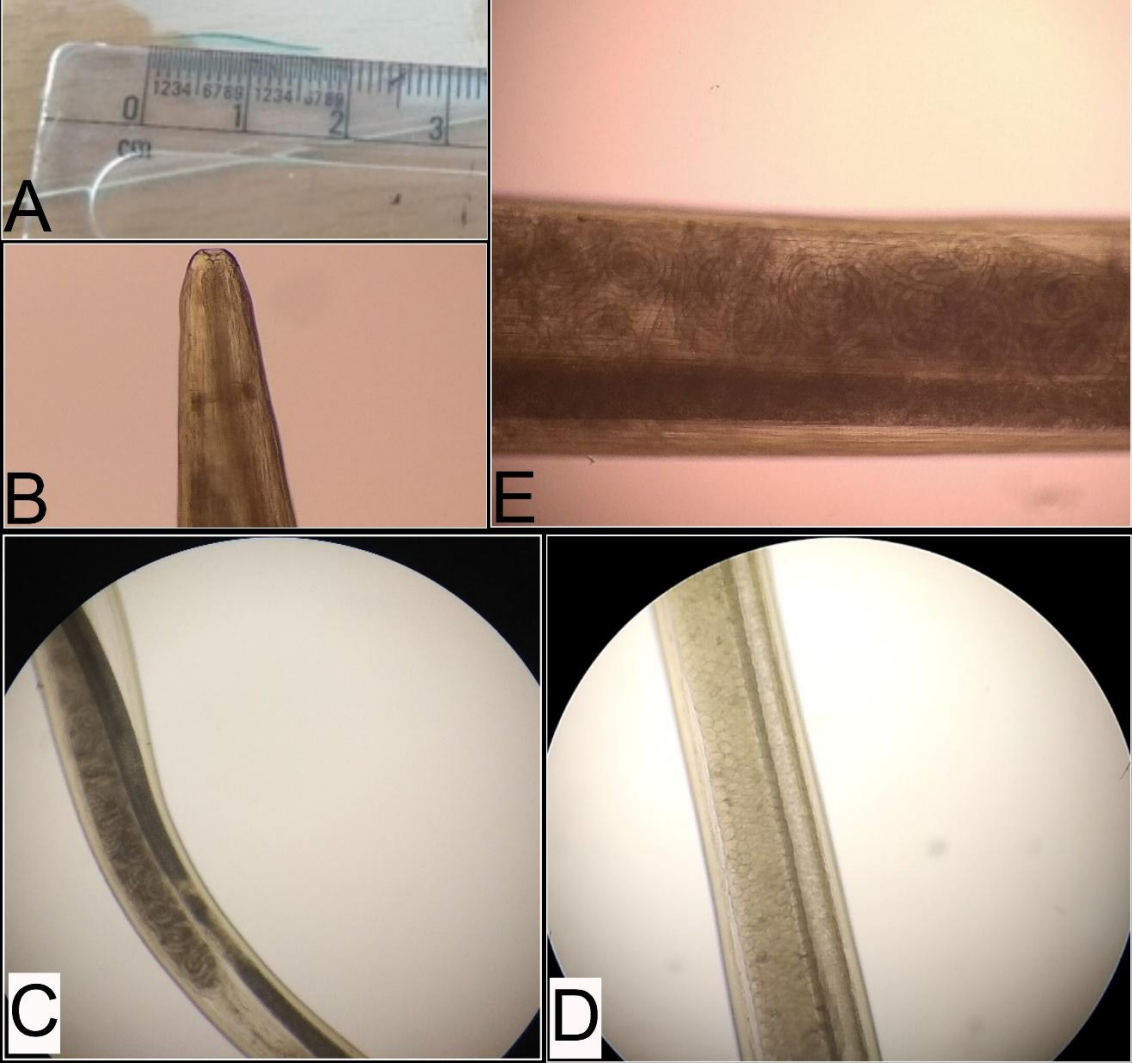
Thelazia measuring approximately 17.0-18.5 mm (1A), Anterior end of Thelazia showing buccal cavity (1B), Middle portion of the worm showing distinct alimentary canal and reproductive system (1C), Proximal uterus of a gravid female of Thelazia showing numerous embryonated eggs (1D), Distal uterus of a gravid female of Thelazia showing coiled first-stage larvae (1E).

All the worms were observed under a microscope after a wet mount preparation. All the worms were creamy white with striations throughout the body. They had a slender body and measured 17.0-18.5 mm in size. Microscopic examination of worm revealed buccal cavity in the anterior part. The alimentary and reproductive systems were well distinguishable from each other. The proximal uterus contained numerous embryonated eggs and the distal uterus contained numerous coiled firststage larvae (Figure 1).

The worms were identified as gravid females of Thelazia based upon the morphological features, and presence of egg and larva inside the reproductive canal of the worms. But the species of Thelazia could not be identified due to the unavailability of a proper parasitological setup.

Ocular symptoms resolved rapidly after the removal of the worms. The patient was instructed to follow-up in 2 weeks and was prescribed with tobramycin ophthalmic drop to prevent secondary bacterial infections. But the patient did not come for follow-up.

## DISCUSSION

Of more than 10 species of Thelazia identified only the species Thelazia callipaeda (Oriental eye worm), Thelazia californiensis (California eye worm), and Thelazia gulosa have been reported to cause human thelaziasis.^1,5,6^ T. callipaeda can be found in China, India, Thailand, Japan, Korea, Russia, northern Europe, and southern Italy while T. californiensis and T. gulosa are solely reported from the United States.^4,5,7^ Most of the cases of human ocular thelaziasis in Asia are caused by Thelazia callipaeda so in the present case T. callipaeda is the most likely species.

The natural vectors of T. callipaeda are believed to be houseflies and Drosophila.^8,9^ These vector flies act as an intermediate host and ingests embryonated eggs or primary-stage larvae along with the conjunctival secretions of definitive hosts. In about 3 weeks, the primary-stage larva matures into an infective third-stage larva inside the body cavity of the flies. While feeding in the eye of a new definitive host the larva emerges from the vector fly and enters the conjunctival sac of the host eye where it matures into an adult in about one month and two molts. The adult females release eggs and the life cycle continues.^1,6,7,10^

Clinical manifestations with thelaziasis range from a foreign body sensation, ocular pruritis, lacrimation, epiphora, exudative conjunctivitis, or corneal edema to keratitis and corneal ulceration in severe cases leading to blindness.^1,7^

Human thelaziasis, like other ocular parasitic infections, is more common in poor socio-economic and rural communities where people live in close proximity with animals.^1,11^ As per the history provided by the mother in the present case, pigs were reared in the neighborhood. Due to the rearing of the pigs, it could have attracted various flies near the house along with the vector for Thelazia.

The natural vector cycle of ocular thelaziasis has a seasonal distribution between July and August.^9^ The presence of a gravid female suggested the presence of a male worm which might have been rubbed out of the eye before symptoms occurred.

Mechanical removal of the worms is sufficient to resolve the symptoms,^2^ yet topical antibacterial ophthalmic drugs like tobramycin are prescribed to prevent secondary bacterial infections that can lead to worsening of the eye conditions which may also lead to ocular opacity to blindness.^1,12^

Although human thelaziasis is a rare occurrence, the present case report warrants the prevalence of thelaziasis in low-socioeconomic families in rural Nepal. As human thelaziasis cases are being reported throughout the world, ophthalmologists should consider the possible Thelazia infection in cases where a patient complains of foreign body sensation, excessive lacrimation, or some black shadow moving in the front eyes which are suggestive of parasitic infestation rather than bacterial or viral infections. The treatment is to carefully remove the worm from the eye which can be easily done by health personnel in the rural health care center with limited resources.

Thelaziasis is transmitted to humans by flies of the genus Drosophilidae thus, primarily, control of the flies and then secondarily, preventive measures like use of bed nets and sanitary habits are critical for the prevention and spreading of the disease.
